# Study Protocol for Teen Inflammation Glutamate Emotion Research (TIGER)

**DOI:** 10.3389/fnhum.2020.585512

**Published:** 2020-10-19

**Authors:** Johanna C. Walker, Giana I. Teresi, Rachel L. Weisenburger, Jillian R. Segarra, Amar Ojha, Artenisa Kulla, Lucinda Sisk, Meng Gu, Daniel M. Spielman, Yael Rosenberg-Hasson, Holden T. Maecker, Manpreet K. Singh, Ian H. Gotlib, Tiffany C. Ho

**Affiliations:** ^1^Department of Psychology, Stanford University, Stanford, CA, United States; ^2^Center for Neuroscience, University of Pittsburgh, Pittsburgh, PA, United States; ^3^Department of Psychology, Yale University, New Haven, CT, United States; ^4^Department of Radiology, Stanford University, Stanford, CA, United States; ^5^Department of Electrical Engineering, Stanford University, Stanford, CA, United States; ^6^Human Immune Monitoring Center, Stanford University, Stanford, CA, United States; ^7^Department of Microbiology and Immunology, Stanford University, Stanford, CA, United States; ^8^Department of Psychiatry and Behavioral Sciences, Stanford University, Stanford, CA, United States; ^9^Weill Institute for Neurosciences, University of California, San Francisco, San Francisco, CA, United States; ^10^Department of Psychiatry and Behavioral Sciences, University of California, San Francisco, San Francisco, CA, United States

**Keywords:** adolescence, depression, anterior cingulate cortex, magnetic resonance spectroscopy, glutamate

## Abstract

This article provides an overview of the study protocol for the Teen Inflammation Glutamate Emotion Research (TIGER) project, a longitudinal study in which we plan to recruit 60 depressed adolescents (ages 13–18 years) and 30 psychiatrically healthy controls in order to examine the inflammatory and glutamatergic pathways that contribute to the recurrence of depression in adolescents. TIGER is the first study to examine the effects of peripheral inflammation on neurodevelopmental trajectories by assessing changes in cortical glutamate in depressed adolescents. Here, we describe the scientific rationale, design, and methods for the TIGER project. This article is intended to serve as an introduction to this project and to provide details for investigators who may be seeking to replicate or extend these methods for other related research endeavors.

## Background

Depressive disorders, including Major Depressive Disorder (MDD), are the leading cause of disability worldwide, costing over $200 billion per year in the United States alone (Greenberg et al., 2015). This burden falls disproportionately on youth: by the age of 18, approximately 25% of individuals in the United States will experience a depressive episode (World Health Organization [WHO], 2017). The onset of depression during adolescence is known to adversely affect the course and prognosis of the disorder. Adolescent-onset depression is associated with longer, more severe, and more recurrent depressive episodes that are often refractory to treatment (Dunn and Goodyer, 2006; Fergusson et al., 2007; Naicker et al., 2013). Indeed, estimates of the probability of recurrence of depression are as high as 60% by 1 year (Birmaher and Arbelaez, 2002; Costello et al., 2002; Birmaher and Brent, 2007; Curry et al., 2011). Because adolescence is a period of significant neurodevelopment, the adverse impact of depression on ongoing brain maturation may explain, in part, why adolescent-onset depression is associated with more severe symptoms and a higher likelihood of recurrence than is adult-onset depression (Naicker et al., 2013).

Adolescence is a period characterized by major life transitions and heightened stress; stress is a frequent precipitant of MDD, a contributor to the course and maintenance of depressive symptoms, and a driver of neuroplasticity (Hammen, 2005; Ho, 2019). Moreover, depression itself is a stressful experience that may affect neurodevelopment through various psychobiological pathways. However, despite the consensus that stress affects neurodevelopmental processes (McEwen, 2012; Nelson and Gabard-Durnam, 2020), and that neurodevelopmental processes should be considered explicitly in models of adolescent MDD (Cicchetti and Rogosch, 2002; Lichenstein et al., 2016; Luby et al., 2016), we still know little about precisely how stress affects adolescent neurodevelopment in ways that increase risk of depression. Addressing this gap in knowledge requires research that takes a longitudinal approach to characterizing trajectories of brain development and depressive symptoms (Gotlib and Ordaz, 2016). In addition to the scarcity of longitudinal neuroimaging data on this topic, another major factor that limits our understanding of these processes is the lack of comprehensive integration of information across different modalities and neurobiological systems that are relevant to the study of adolescent stress, neurodevelopment, and depression.

Investigators have posited that inflammatory processes are critical in understanding how stress affects neurodevelopment in the context of depression (Miller et al., 2009; Ménard et al., 2016). It is well recognized that life stress activates the immune system by initiating a cascade of inflammatory responses, including increased production of pro-inflammatory cytokines in the peripheral nervous system (Miller et al., 2009). Critically, peripheral cytokines can cross the blood-brain barrier and alter its permeability (Banks et al., 2009), leading to increased inflammatory signaling in the central nervous system through the kynurenine pathway. Specifically, inflammatory cytokines activate the enzyme indoleamine-2,3 dioxygenase, which converts tryptophan (a precursor to serotonin) to kynurenine (Guillemin et al., 2001; Dantzer, 2017); a metabolite from this kynurenine pathway is quinolinic acid, which is a *N*-methyl-*D*-aspartate (NMDA) receptor agonist. The binding of quinolinic acid to NMDA receptors stimulates the production of glutamate; simultaneously, the inflammatory cascade initiated by the passage of peripheral cytokines into the central nervous system compromises the structural integrity of astrocytes and their ability to reuptake glutamate, as well as leads to damage of oligodendrocytes, the glial cells that produce myelin (Káradóttir and Attwell, 2007; Miller et al., 2009; Matute, 2011; Miller and Raison, 2016; Haroon et al., 2017). Thus, glutamatergic excitotoxicity through the kynurenine pathway and related pathways may be a key mechanism by which stress leads to the neurophenotypes commonly identified in depression, including gray matter loss (as measured by smaller limbic volumes, cortical thinning, and lower surface area), lower integrity of white matter microstructure in fronto-cingulate-limbic tracts (Aghajani et al., 2014; Bessette et al., 2014; LeWinn et al., 2014), and altered intrinsic fronto-cingulate-limbic connectivity (Connolly et al., 2013; Ho et al., 2014, 2015, 2017b; Kerestes et al., 2014). While neuroinflammation and mediators of the immune response in the central nervous system, such as microglia, cannot currently be measured non-invasively, technologies such as proton magnetic resonance spectroscopy (^1^H-MRS) can be used to measure non-invasively the downstream effects of inflammation on neurotransmitter levels, including glutamate.

A number of studies using proton magnetic resonance spectroscopy (^1^H-MRS), which permits non-invasive *in vivo* imaging of neurometabolites, have found reduced concentrations of glutamate or Glx (the sum of glutamate and glutamine, which is a proxy of glutamate metabolism as this signal is dominated by Glu) in multiple brain regions of depressed adults, including the anterior cingulate cortex (ACC) and medial frontal cortex (for a review, see Yüksel and Öngür, 2010; Sanacora et al., 2012; Moriguchi et al., 2019). Interestingly, these findings stand in contrast to the work reviewed above, which have consistently found *elevated* levels of Glu in response to stress and *higher* levels of Glu contributing to neurotoxicity (Miller et al., 2009; Miller and Raison, 2016; Haroon et al., 2017). Given that the vast majority (85.7%) of studies in the field have been in adults and not adolescents (Moriguchi et al., 2019), one possibility is that younger individuals with MDD exhibit higher levels of Glu in the ACC (and other cortical regions), but that over time, this results in depression-related cortical atrophy and, ultimately, reduced Glu compared to healthy controls. It is also important to note that with the exception of the study by Gabbay et al. (2017), which assessed 44 depressed adolescents, all of the prior MRS studies in depressed adolescents examining Glu (as described in Moriguchi et al., 2019) recruited relatively small sample sizes of depressed adolescents (*n* < 20), did not examine associations with inflammatory cytokines, and did not utilize longitudinal study designs to assess neurodevelopmental trajectories.

Indeed, no study to date has examined inflammation and its effects on glutamate levels and neurodevelopmental trajectories in adolescents with depression. Addressing this gap will help to elucidate whether (1) elevated inflammation and glutamate are already present in depressed adolescents or, alternatively, are consequences of chronic depression, as has been seen in adults with treatment-resistant depression; and (2) higher levels of inflammation and glutamate affect adolescent neurodevelopment in ways that increase risk for the development of subsequent episodes of depression. Moreover, researchers have posited that antioxidants, including glutathione and ascorbate, buffer against the neurotoxic effects of excessive glutamate in neurons, and may serve a neuroprotective role against glutamatergic excitotoxicity (Ballaz et al., 2013). That is, antioxidants may attenuate the effects of inflammation on glutamate, as well as the effects of inflammation-related glutamatergic neurotoxicity on neurodevelopment and depressive symptoms; these formulations, however, have not yet been tested.

**FIGURE 1:**
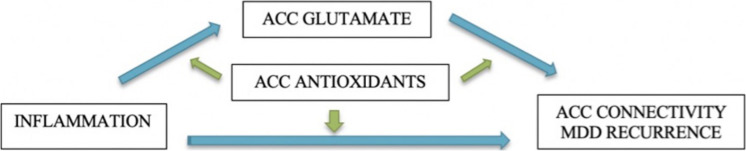
Conceptual model and study aims. Green arrows indicate moderation of associations.

The Teen Inflammation Glutamate Emotion Research (TIGER) study was developed to address these questions. We are using multimodal imaging to comprehensively assess neurophenotypes of depressive disorders and integrating information across traditionally disparate yet empirically relevant neurobiological systems (i.e., inflammatory and glutamatergic systems) to elucidate how neurodevelopment is affected in adolescents with depression. Specifically, we are testing the central hypothesis that glutamate mediates the links between inflammation and the developmental trajectories of ACC connectivity over 18 months. We focused our investigation on the ACC because it is a large integrative region that spans multiple networks implicated in depression – including the salience network, the default mode network, and the central executive network – and subserves a host of functions commonly disrupted in depression, including emotion generative and regulatory processing, reward encoding, threat detection, learning, and error monitoring (Botvinick et al., 2004; Stevens et al., 2011; Kolling et al., 2016; Shenhav et al., 2016). Critically, the ACC undergoes significant maturation during adolescence (Power et al., 2011), and altered functional and structural connectivity of the ACC is one of the most robust neurophenotypes that has emerged in studies of adolescents with depression (Davey et al., 2012; Connolly et al., 2013; Ho et al., 2015, 2017b; Lichenstein et al., 2016). Specifically, in the TIGER project we seek to address the following questions (see Figure 1):

(1) Does glutamate concentration in the ACC mediate the associations between elevated pro-inflammatory cytokines and depressive neurophenotypes (measured by ACC connectivity)?
(2) Do antioxidants (as measured by glutathione and ascorbate) in the ACC moderate the associations among inflammation, ACC glutamate, and longitudinal trajectories of depressive neurophenotypes (measured by ACC connectivity)?
(3) What are baseline predictors of remission versus recurrence of depression over 18 months?

Finally, we are also recruiting a sample of psychiatrically healthy adolescents in order to examine the possibility that deviations from normative levels or trajectories in these neurobiological markers characterize adolescent depression.

## Methods and Study Design

### Participants

**FIGURE 2:**
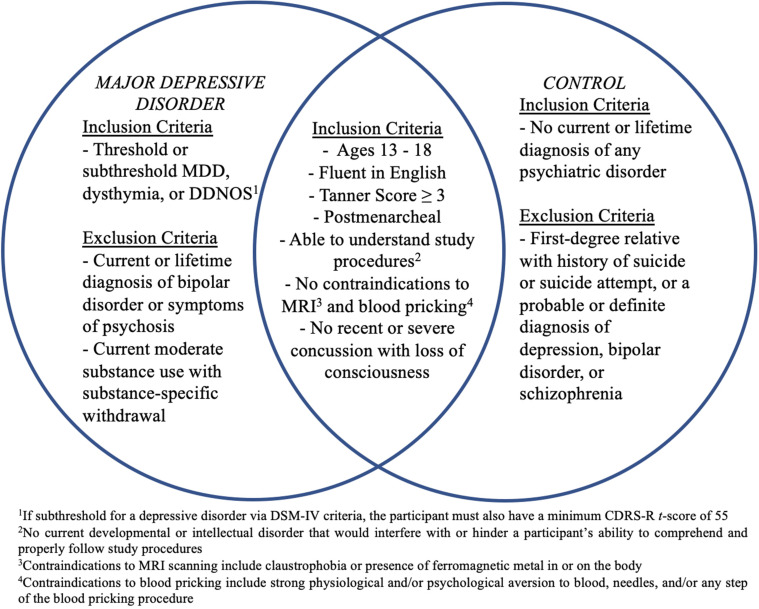
Inclusion and exclusion criteria for participants with Major Depressive Disorder (MDD) and healthy controls. CDRS-R, Children’s Depression Rating Scale-Revised; DDNOS, depressive disorder not otherwise specified; MDD, Major Depressive Disorder; MRI, magnetic resonance imaging.

Participants will be 60 depressed high school-aged adolescents (MDD) (ages 13–18 years) and 30 age- and sex-matched healthy controls (CTL) recruited from the San Francisco Bay Area community. See Figure 2 for a list of inclusion/exclusion criteria for each diagnostic group. Participants will be recruited using flyers; advertisements on Craigslist, Nextdoor, and Facebook; and an internal referral program. The study has been approved by the Institutional Review Boards at Stanford University and the University of California, San Francisco. Participants and their parent(s)/legal guardian(s) will complete written assent and informed consent, respectively, and will be compensated for their participation.

### Design and Procedures

**FIGURE 3:**
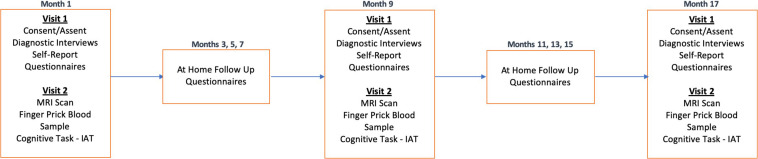
Longitudinal study protocol. TIGER is a longitudinal study approximately 18-months long with 3 timepoints, approximately 9 months apart. Each timepoint will include two laboratory visits (Visit 1 and Visit 2). Every other month between laboratory sessions we will also obtain self-report measures of depression (RADS-2, PHQ-9) and anxiety (MASC-2), as well as information on any potential treatment changes. Please also see Table 1 for a list of measures collected within each session visit at each timepoint.

The study will take place over an 18-month period, with three in-person laboratory sessions for all participants, approximately 9 months apart. Each timepoint (T1, T2, T3) will consist of two in-person visits (V1, V2) that will occur within 3 weeks of one another. T1 (V1) will include diagnostic and clinical interviews with adolescents and their parent(s)/legal guardian(s) to confirm the adolescent’s eligibility, followed by self-report questionnaires. Once an adolescent is confirmed to be eligible, the participant will be invited to attend the second lab session (V2). V2 will include the MRI brain scan and a finger prick blood sample. These procedures will be repeated at T2 and T3. Between each of the three time points, participants will be asked to complete three surveys (2 online and 1 via phone call) from home every other month (e.g., M3, M5, M7, etc.). See Figure 3 for more details.

**TABLE 1:** Summary of primary measures in TIGER.

Method	Measure	Domain assessed	V1	V2	M
Clinical interview:	K-SADS-PL	Clinical diagnosis	X		
	CDRS-R	Depression symptom severity	X		
	C-SSR-S	Suicidal ideation and behaviors	X		
	SITBI	Non-suicidal self-injury	X		
	FIGS*	Family psychiatric history	X		
Self-report:	Tanner	Pubertal development	X		
	RADS-2	Depression symptom severity	X		X
	SIQ-JH	Explicit suicidal ideation	X		
	PHQ-9	Depression symptom severity	X		X
	MASC-2	Anxiety symptom severity	X		X
	MPVS	Bullying	X		
	STRAIN	Life stress	X		
	CTQ*	Childhood trauma	X		
	DERS*	Emotion regulation difficulties	X		
MRI:	T1-weighted SPGR	Grey and white matter		X	
	Resting-state fMRI	Functional connectivity		X	
	Diffusion-weighted MRI	Structural connectivity		X	
	^1^H-MRS (ACC)	Glutamate, Glutathione, Ascorbate		X	
	ACC Glu	Glutamate		X	
	ACC GSH and ACC Asc	Antioxidants		X	
Cognitive task:	IAT – death version	Implicit suicidal ideation		X	
Blood:	IL-1β, IL-6, IL-10, TNF-α	Inflammation		X	

Measures will be assessed at all three timepoints unless marked with a * which indicates the measure will only be administered at T1. Measures under column “M” are administered at the bi-monthly online follow-up assessments.

### Clinical and Behavioral Measures

#### Demographics

Participant age, sex assigned at birth, gender identity, sexual orientation, ethnicity, and race will be obtained through self-report questionnaires, while family socioeconomic status (income range and highest education of parent) will be obtained through parent-report.

#### Family History

To capture family history of psychiatric disorders, with a focus on mood disorders and suicide, in all first-degree blood-relatives of the participant, we will administer a modified version of the Family Interview for Genetic Studies (FIGS) (Gershon et al., 1988) to the adolescent’s participating parent and/or legal guardian. If any endorsements of a down or elevated mood are made, the interviewer will continue into a screener probing for DSM-IV symptom criteria for depressive and bipolar disorders. A diagnosis will then be determined to be not present, suspected, or definite. In addition to asking about family history of depression and suicide, each parent will also be asked general screener questions to capture other significant psychiatric disorders: “*Did any [first degree relative] have any history of any psychiatric disorders or any problems with their nerves, emotions, or substance use? Did any see any professionals for their emotions or have any medications or treatments?*” All other suspected disorders captured in the general screener will be further probed, and information related to symptoms, diagnoses, professional treatment, and impairment will be used to determine whether the disorder was not present, suspected, or definite.

#### Pubertal Development

To measure pubertal development, we will be using self-report Tanner staging (Marshall and Tanner, 1968, 1970; Morris and Udry, 1980), wherein participants select how closely their pubic hair and breast/testes resemble an array of schematic drawings on a scale of 1 (prepubertal) to 5 (postpubertal). Self-report Tanner staging scores, particularly in older adolescents, have been found to be strongly correlated with physicians’ physical examinations (Coleman and Coleman, 2002; Shirtcliff et al., 2009). As in previous studies of depressed adolescents, we will use the average of the pubic hair (i.e., adrenal) and breast/testes (i.e., gonadal) Tanner scores to index overall pubertal development (Ho et al., 2013, 2014, 2016). Female participants will also be asked to provide information on their menstrual cycle by providing approximate dates of their first period, as well their most recent periods at each timepoint. Participants will also be asked to report on details of their current and past use of hormone contraceptives.

#### Depression and Psychiatric History (Clinical Interviews)

Participants and their parent(s)/legal guardian(s) will be administered the Kiddie Schedule for Affective Disorders and Schizophrenia Interview – Present and Lifetime Version (K-SADS-PL) (Kaufman et al., 2000), a semi-structured clinical interview designed to yield reliable and valid diagnoses of current and past history of psychiatric disorders according to DSM criteria. For the present study, we will be assessing present and lifetime history of the following Axis I disorders according to DSM-IV criteria: Depression, Mania (for the purpose of determining exclusion), Psychosis (for the purpose of determining exclusion), Alcohol Abuse and Dependence (for the purpose of determining exclusion), Panic Disorder, Social and Specific Phobia, Generalized Anxiety Disorder, Obsessive Compulsive Disorder, Conduct Disorder, Oppositional Defiant Disorder, Disruptive Mood Dysregulation Disorder, and Post-Traumatic Stress Disorder. We will also be evaluating Attention Deficit Hyperactivity Disorder (ADHD) and Substance Use Disorders according to DSM-V criteria. The decision to rely on DSM-V criteria for ADHD was due to the later age cutoff for symptom presentation (age 12 versus age 7). The decision to rely on DSM-V criteria for substance use was due to the inclusion of substance-specific withdrawal symptoms. Moreover, there is compelling evidence across adolescents and substances that criteria for abuse and dependence in the DSM-IV reflect the same underlying condition rather than distinct nosologies (Hasin et al., 2013). Within each psychiatric module, ratings on a scale of 0 to 3 (no information to threshold) will be assigned to both the current and the most severe past episode. Age of onset, duration of episode, and number of episodes (if relevant) will be assessed for each disorder, as well as any academic, social, and familial impairment. To assess whether the participant has experienced remission or recurrence of a depressive disorder at T2 and T3, we will follow procedures from the Treatment for Adolescents with Depression Study (TADS) (Curry et al., 2011), and define recurrence as experiencing a clinically significant episode of depression following recovery (i.e., remission lasting a minimum of 8 weeks).

Additionally, to derive dimensional scores of depression severity, we will administer the Children’s Depression Rating Scale-Revised (CDRS-R) (Poznanski and Mokros, 1996) to all participants and their parents. The CDRS-R is a clinician-rated scale and one of the most widely used rating scales for assessing severity and change of depression symptoms for clinical research trials in pediatric depression (Isa et al., 2014). The CDRS-R is comprised of 17 questions, the first 14 of which are administered to both parent and adolescent participants, while the last three items are rated based on clinician observation and assess non-verbal characteristics such as depressed affect, listless speech, and hypoactivity. Interviewers will assign a total summary score integrating both parent and child interviews. Participants who are subthreshold for depressive disorders based on the K-SADS-PL but have a CDRS-R *t*-score ≥ 55 will be included (see Figure 2).

#### Depression (Self-Report)

Self-reported severity of symptoms will be assessed with the Reynolds Adolescent Depression Scale (RADS-2) (Reynolds, 2010), a 30-item questionnaire designed to assess severity of depressive symptoms in adolescents in both school and clinical settings (Reynolds, 2002). The RADS-2 was validated in an ethnically diverse North American sample of adolescents ages 11–20 years. In addition to a total severity score, the RADS-2 also generates scores on four specific dimensions of depression: *Dysphoric Mood*, *Anhedonia/Negative Affect*, *Negative Self-Evaluation*, and *Somatic Complaints*. We anticipate that data from the RADS-2 subscales will be useful for exploring hypotheses concerning subtypes of depression.

Finally, we will also administer the Patient Health Questionnaire-9 (PHQ-9), which is a nine-item questionnaire frequently used in both clinical and research settings to assess depression severity and symptom change (e.g., in response to treatment) in both adolescents and adults (Kroenke et al., 2001). We anticipate that data from the PHQ-9 will facilitate opportunities to combine our data with other studies across developmental populations.

#### Anxiety (Self-Report)

To assess symptoms of anxiety, we will administer the Multidimensional Anxiety Scale for Children (MASC-2) (March, 2012), a 39-item questionnaire which generates four subscales—*Social Anxiety*, *Separation Anxiety*/*Panic*, *Harm Avoidance*, and *Physical Symptoms*—based on six symptom domains: tense/restless, somatic/autonomic, perfectionism, anxious coping, humiliation/rejection, and performance fears. The MASC-2 has high convergent validity, both strong inter-rater and test–retest reliability, and has been widely used to assess anxiety within adolescents (March et al., 1997; Wei et al., 2014).

#### Suicidal Thoughts and Behaviors (Clinical Interviews)

Because depression is one of the strongest psychiatric risk factors for suicidal thoughts and behaviors (STBs) (Franklin et al., 2017; Ribeiro et al., 2018; Melhem et al., 2019), and because adolescence is a time when STBs rise (Nock et al., 2013), we will utilize multiple measures designed to assess STBs in adolescents, all of which are part of the Suicide Specialty Collection of the PhenX Toolkit^1^.

Within the depression module of the K-SADS-PL, we will assess various levels of STBs – ranging from abstract thoughts, recurrent thoughts, suicidal ideation, suicidal acts (researching methods, obtaining materials, creating a plan), and suicide attempts. To supplement information from the K-SADS-PL and to further classify suicidal ideation and behavior, we will administer the pediatric version of the Columbia Suicide Severity Rating Scale (C-SSRS) (Posner et al., 2008). The C-SSRS is a semi-structured interview and has strong validity, internal consistency and high sensitivity (Posner et al., 2011; Gipson et al., 2015). Through administration of the C-SSRS, interviewers will assess the severity of suicidal ideation and gather information regarding the frequency, duration, and controllability of thoughts; possible deterrents to acting; and reasons for ideation. Interviewers will also utilize the C-SSRS to gather information about suicidal behaviors, including details regarding actual, interrupted, or aborted attempts as well as preparatory acts such as gathering materials or writing a suicide note.

Finally, given the co-occurrence of suicidal thoughts and attempts with non-suicidal self-injurious (NSSI) behaviors (Nock et al., 2006; Asarnow et al., 2011; Guan et al., 2012; Klonsky et al., 2013), we will also administer portions of the Self-Injurious Thoughts and Behavior Interview (SITBI) (Nock et al., 2007) to assess suicidal gestures and NSSI. The SITBI is a structured interview that assesses the history, frequency, and intensity of thoughts and behaviors related to non-suicidal self-injury. Ages of onset, as well as frequency of thoughts and behaviors in the last year (or since last visit, for T2 and T3 assessments), last month, and week prior to the assessment will also be obtained. Additionally, the SITBI includes questions assessing medical attention received for NSSI behaviors, as well as participants’ perception that these NSSI thoughts and behaviors are likely to recur. The SITBI has high convergent validity, both strong inter-rater and test–retest reliability, and has been widely used with adolescents (van Alphen et al., 2017; Stewart et al., 2019; Vergara et al., 2019).

#### Suicidal Ideation (Self-Report)

To measure severity and frequency of suicidal ideation in the past month, we will administer the Suicidal Ideation Questionnaire–Junior High version (SIQ-JH) (Reynolds, 1987). The SIQ-JH is a 15-item measure designed for use with adolescents between the ages of 12–18 years, and has high internal consistency, test–retest reliability, and predictive validity (Reynolds, 1987).

#### Implicit Suicidal Ideation (Cognitive Task)

In addition to explicit or self-disclosed methods of suicidal ideation, we will administer a computerized task to probe suicidal ideation without relying on self-report, as there may be several reasons why an adolescent may not be truthful about their thoughts surrounding death or suicide (Nock et al., 2010). Specifically, we will be using the death-version of the implicit association test (IAT), which is a 5-min computerized task that will allow us to measure participants’ response latencies to associations between self/not self-related stimuli and life/death-related stimuli to approximate the strength of each individual’s association with self and death (Nock et al., 2010). The IAT has been shown to have strong reliability (Cunningham et al., 2001; Nosek et al., 2005), construct validity (Lane et al., 2007), and sensitivity to clinical change in treatment (Teachman and Woody, 2003), and has also been used to predict severity of future suicidal ideation, non-suicidal self-injury, and attempt in adolescents (Cha et al., 2016; Glenn et al., 2019). Importantly, we have previously demonstrated in an independent community sample of adolescents (ages 9–13 years) that morphological alterations in the dorsal striatum – specifically the putamen and caudate – predict IAT bias scores 2 years later (Ho et al., 2018). Interestingly, putamen and caudate volumes did not predict SIQ-JH scores, suggesting that not only are dopaminergic striatal structures implicated in suicide risk, but also that neurobiological phenotypes may be better predictors of *objective* markers of suicide risk.

#### Life Stress

Given the key role of stress in our theoretical model, we will obtain comprehensive information on cumulative life stress in each participant using the Adolescent version of the Stress and Adversity Inventory (STRAIN) (Slavich et al., 2019). The Adolescent STRAIN is a self-administered computerized set of questions probing exposure to 75 distinct stressors, including 33 acute life events and 42 chronic difficulties. Through branching logic, additional questions are generated to assess the severity, frequency, timing, and duration of any endorsed stressor. The STRAIN also separates stressors occurring across different life domains (e.g., education, health) into five mutually exclusive types based on gold-standard stress assessments (Slavich and Shields, 2018): *Interpersonal Loss* (e.g., experiences characterized by the dissolution of relationships), *Physical Danger* (e.g., life-threatening circumstances, such as being robbed), *Entrapment* (e.g., circumstances that are difficult to escape, such as caring for a sibling with a disability), *Humiliation* (e.g., experiences characterized by social rejection, such as being mocked publicly), *Role Change*/*Disruption* (e.g., experiences marked by major life transitions, such as starting high school). Although the test–retest reliability of the Adolescent STRAIN has not yet been examined, its parent instrument, the Adult STRAIN, has been shown to exhibit excellent test–retest reliability (Slavich and Shields, 2018). More details on the psychometric properties of the Adolescent STRAIN can be found in Slavich et al., 2019.

#### Childhood Trauma Questionnaire

Meta-analytic and epidemiological evidence has shown that maltreatment experienced during childhood is significantly associated with depression in adolescents (McLaughlin et al., 2012; Humphreys et al., 2020; LeMoult et al., 2020), consistent with the formulation that adversity experienced during sensitive periods of development has an outsized effect on subsequent neurodevelopmental trajectories supporting mental health (Nelson and Gabard-Durnam, 2020). To measure adversity experienced during childhood, we will use the Childhood Trauma Questionnaire Short Form (CTQ-SF) (Bernstein et al., 2003), which is one of the most widely used measures of childhood maltreatment. The CTQ-SF is a reliable and empirically validated 28-item version of the original 70-item questionnaire (see Bernstein et al., 1994), and is rated on a 5-point scale. Along with a total score indexing severity of childhood trauma, the CTQ-SF has five clinical scales assessing childhood abuse and neglect: *Emotional Abuse*, *Physical Abuse*, *Sexual Abuse*, *Emotional Neglect*, and *Physical Neglect*. For readability, we administered an adapted version of the CTQ-SF in which we excluded the three-item validity scale, shortened item descriptions, and revised the directionality of reverse-scored items, which were neglect-related items only (e.g., “*There was someone in my family who helped me feel like I was important or special*” to “*I was never made to feel important*”).

#### Peer Victimization

Since peer victimization and bullying have been linked with depressive symptoms in adolescents (Sweeting et al., 2006; Hong and Espelage, 2012; Stapinski et al., 2014), we also will administer the Multidimensional Peer Victimization Scale (MPVS) (Mynard and Joseph, 2000) to assess peer-related abuse. The MPVS is a self-report questionnaire comprised of 16 items that assess the frequency with which a child experienced certain types of peer victimization and manipulation in the last school year. Along with a total score indexing severity of peer victimization, the questionnaire generates four subscales: *Physical Victimization*, *Social Manipulation*, *Verbal Victimization*, and *Attacks on Property*. The MPVS has strong internal consistency, split-half reliability, and concurrent, convergent, and discriminant validity (Joseph and Stockton, 2018). Finally, to complement the MPVS, we also will query participants about recent experiences of bullying using an in-house measure. Participants will report whether or not they are currently being bullied, and if so, will describe who is bullying them (e.g., kids at school, in their grade or in class, in their neighborhood, family members), how many individuals are bullying them, and how they are being bullied.

#### Emotion Regulation

Given ample evidence that emotion regulation dysfunction is associated with the onset and recurrence of depression (Silk et al., 2003; Kuyken et al., 2006; Burwell and Shirk, 2007), we assessed emotion regulation strategies using the Difficulties in Emotion Regulation Scale (DERS) (Gratz and Roemer, 2003). The DERS is a 36-item measure which assesses emotion regulation tendencies and includes six subscales that assess different aspects of emotion regulation such as understanding, awareness and related behaviors. DERS has strong reliability and validity with adolescents (Weinberg and Klonsky, 2009; Neumann et al., 2010; Sarıtaş-Atalar et al., 2015) and adults (Orgeta, 2009; Kökönyei et al., 2014; Kaufman et al., 2016). Because the DERS was designed to assess trait level emotion regulation strategies (Hallion et al., 2018), we will only administer the DERS at T1.

### Neuroimaging

All participants will complete the following MRI scans on a 3T Discovery MR750 (GE Medical Systems, Milwaukee, WI, United States) with a 32-channel head coil (Nova Medical, Wilmington, MA, United States) at the Stanford Center for Cognitive and Neurobiological Imaging. See Table 2 for detailed acquisition parameters for each scan.

**TABLE 2:** Summary of scan sequences and parameters.

	Voxel size (isometric mm, unless otherwise specified)	Number of slices (orientation)	FOV (cm)	TR/TE/TI (ms)	Number of volumes	Flip angle (°)	Duration (min:sec)
T1-weighted MRI	1.0	156 (axial)	25.6	8.2/3.2/600	N/A	12	3:40
Optimized PRESS (dACC)	Variable, not isometric	1 (oblique)	24.0	2000/35/NA	N/A	90	5:04
Optimized PRESS (rACC)	Variable	1 (axial)	24.0	2000/25/NA	N/A	90	5:04
Resting-state BOLD EPI	2.9	42 (oblique)	23.2	2000/30/NA	240	77	8:00
Resting-state fieldmap	2.9	42 (oblique)	22.4	700/4.5/NA	N/A	54	0:27
Diffusion-weighted MRI	2.0	64 (axial)	24.0	8500/94.2/NA	66 (60 *b* = 2000, 6 *b* = 0)	90	9:30
Diffusion-weighted MRI fieldmap	2.0	64 (axial)	24.0	700/4.5/NA	N/A	54	0:27
Quantitative T1	2.0	25 (axial)	24.0	3000/25/50	31	77	2:03 per scan (second scan with reverse phase-encoding)

#### T1-Weighted Anatomical MRI

A set of high-resolution T1-weighted anatomical images will be acquired using GE’s “BRAVO” scan, which is a fast spoiled gradient-recalled (SPGR) sequence with parameters tuned to optimize tissue contrast. In addition to using images from this scan for prescribing voxels in the MRS scan (see below) and facilitating registration with the other images we will collect, we will also preprocess all T1-weighted MRI data using FreeSurfer 6.0 (Fischl et al., 2002)^2^ to perform tissue segmentation and estimate subcortical gray matter volumes, cortical thickness, and surface area according to Desikan atlas (Desikan et al., 2006). Each segmentation will be manually checked for quality assurance according to the protocols established by the global consortium, Enhancing NeuroImaging Genetics through Meta-Analysis (ENIGMA)^3^. Outliers, defined as three absolute standard deviations away from the sample mean, will be flagged for additional visual inspection. Any structure that is poorly segmented will be excluded from further analysis only after careful visual inspection.

#### Magnetic Resonance Spectroscopy

To estimate concentrations of glutamate (Glu), glutathione (GSH), and ascorbate (Asc), we will use a proton magnetic resonance spectroscopy (^1^H-MRS) scan based on a modification of the GE Healthcare PRESS product sequence, PROBE-p^TM^. Two features were added to the product PROBE-p^TM^ sequence for improved localization: (1) 16 step phase cycling (EXORCYCLE on the two refocusing RF pulses) and (2) application of a sensitive point echo planar (EP) waveform during acquisition to further eliminate out-of-slice artifact in the logical z direction (Bodenhausen et al., 1977; Webb et al., 1994; Gu et al., 2018). To avoid suppression of Asc at 4.1 ppm, the bandwidth of the CHESS RF water suppression pulses will be reduced from 150 to 75 Hz. At T1, the location of the MRS voxel for the dorsal ACC (and rostral ACC), will be graphically prescribed manually following construction of the 3D T1-weighted anatomical scan (see above) using anatomical landmarks. At T2 and T3, we will use an automated voxel placement tool that uses non-linear warping between current native subject space and prior native subject space (i.e., at T1) to identify precise voxel locations in scanner space in real-time during data acquisition and to minimize bias in manual prescription^4^.

All spectra of interest will be quantified using LCModel (Provencher, 2001; see Table 3 for more details). Experimental GSH and Asc basis spectra will be acquired from custom built 50 mM GSH and Asc spherical phantoms with pH of 7.2 at 37°C in an otherwise synthetic basis set to improve accuracy (due to the complexity of the GSH and Asc resonances and their dependence on temperature, simulated basis spectra are often inaccurate). All metabolite concentration levels will be expressed as ratios to total creatine (i.e., sum of creatine and phosphocreatine) levels (which are relatively high and stable across different tissue types of the brain, and thus, often used as an internal reference standard for characterizing other spectra). In addition to quantifying concentrations of Glu (and GSH and Asc), because of the dynamic cycling between glutamine (Gln) and Glu (extracellular Glu is converted into Gln in astrocytes before being released and taken up by neurons where it is synthesized into Glu or GABA; Ramadan et al., 2013), we will also consider the total sum of Glu and Gln (Glx), and the ratio of Glu to Gln as complementary indices of glutamate metabolism. Finally, because *N*-acetylaspartate (NAA) is present almost exclusively in neurons and is widely considered a marker of neuronal health, whereas myo-inositol (mI) and choline (Cho) are found mostly in glial cells (Brand et al., 1993; Griffin et al., 2002), we will also explore these neurometabolites in analyses specific to gray and white matter, respectively. We will use the Cramér–Rao lower bounds (CRLB), a measure of the reliability of the fit, with a quality criterion set at <35% for each individual metabolite. Tissue segmentation (e.g., percentage of gray matter) in prescribed voxels will also be used as a potential covariate.

**TABLE 3:** Metabolites of interest imaged by magnetic resonance spectroscopy.

Metabolite	Abbreviation
Creatine	Cr
Glutamate	Glu
Glutathione	GSH
*N*-Acetylaspartate	NAA
Ascorbate	Asc
Glutamine	Gln
Glutamate + Glutamine	Glx
myo-Inositol	mI
Choline (Glycerophosphocholine + Phosphocholine)	Cho (GPC + PCH)
Creatine + Phosphocreatine	Cr + PCr

#### Resting-State fMRI

**FIGURE 4:**
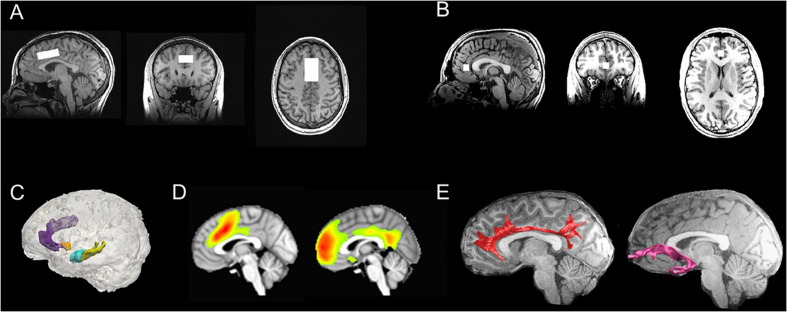
Multimodal neuroimaging scans in TIGER. **(A)** Representative dorsal ACC voxel from the MRS scan; **(B)** Representative rostral ACC voxel from the MRS scan; **(C)** Representative FreeSurfer-based segmentations of the dorsal ACC (light purple), rostral ACC (dark purple), striatum (orange), hippocampus (yellow), and amygdala (blue); **(D)** Group-level computations of the salience network anchored in the dorsal ACC (left) and default mode network anchored in the medial prefrontal cortex/rostral ACC and cingulate gyrus (right) derived from resting-state fMRI data using ICA (all maps thresholded at *t*_68_ > 3.93; α = 0.0001); **(E)** Representative tractography of the cingulum bundles (red) and uncinate fasciculus (pink), which are two major white matter tracts that connect fronto-cingulate-limbic structures. ACC, anterior cingulate cortex; ICA, independent components analysis; MRS, magnetic resonance spectroscopy; TIGER, Teen Inflammation Glutamate Emotion Research.

To examine intrinsic functional connectivity, we will acquire resting-state T2^∗^-weighted BOLD fMRI data using an echo planar image (EPI) sequence. Resting-state fMRI data will be pre-processed using standard methods in AFNI and FSL that we have implemented based on previous work from our group (Connolly et al., 2013, 2017; Ho et al., 2015, 2017b; Sacchet et al., 2016; Ordaz et al., 2018; Schwartz et al., 2019). Specifically, EPI timeseries data will be despiked, slice-time and motion corrected, then subsequently aligned to the T1-weighted images using a local Pearson correlation method (Saad et al., 2009), and spatially smoothed. We will then submit the pre-processed data into a voxelwise multiple linear regression to regress out effects from non-brain and physiological processes (mean signal from WM and CSF, 6 motion parameters at each time point, 6 motion parameters at the prior time point, and the square of each of these 12 motion parameters); the resulting residuals will then be bandpass filtered (0.009 < *f* < 0.08 Hz), demeaned, and used for subsequent analyses. For the purposes of testing study aims, we will conduct group-level independent components analysis (ICA) followed by dual regression. Group ICA is a data-driven multivariate signal-processing method that spatially clusters fMRI data based on the strength of their temporal correlations (Kiviniemi et al., 2003; Beckmann et al., 2009); when followed by dual regression, this approach has higher test–retest reliability than seed-based functional connectivity (Zuo et al., 2010; Smith et al., 2014). Based on previous work from our group and others implicating dorsal and rostral ACC connectivity in adolescent depression (Ho et al., 2015, 2017b; Lichenstein et al., 2016), we will focus on connectivity of a component of the salience network, which is anchored in the dorsal ACC and enjoys robust connectivity with the anterior insula and subcortical structures (Seeley et al., 2007; Menon and Uddin, 2010; Power et al., 2010), and a component of the default mode network, which includes rostral ACC, medial prefrontal cortex, and the posterior cingulate cortex (Andrews-Hanna et al., 2010). See Figure 4 for more details.

#### Diffusion-Weighted MRI

Diffusion-weighted (DW) MRI data will be acquired using a modified version of the GE Healthcare DW-EPI sequence with dual spin echo scans, where the polarity of the second 180° pulse is inverted relative to the first 180°, thereby causing off-resonance signal from fat to get defocused and reducing fat-shift artifacts (Reese et al., 2003; Sarlls et al., 2011). Given evidence in the literature implicating the cingulum bundles, which are major white matter tracts connecting ACC with prefrontal cortex (cingulum cingulate), along with the ACC and hippocampus (cingulum hippocampus), and the uncinate fasciculus (a major white matter tract connecting the amygdala with prefrontal cortex) with depression in adolescents (LeWinn et al., 2014; Lichenstein et al., 2016), we will focus our analyses on white matter microstructure of these fronto-cingulate-limbic tracts, with a specific focus on tracts that cross the ACC. Specifically, we will acquire diffusion-weighted MRI (dMRI) sequence using an echo planar imaging sequence with isotropic voxels sampling 96 directions. All dMRI data will be pre-processed (e.g., registration, eddy-current and motion correction, resampling) based on VISTA tools^5^ that have been implemented by our team previously in adolescents (Ho et al., 2017a). Diffusion tensors will be fit using least squares to generate voxelwise fractional anisotropy (FA) maps. Whole brain deterministic fiber tracking will be performed with Automated Fiber Quantification (AFQ)^6^. The seeds for tractography will be selected from a uniform 1 mm 3D grid spanning the whole brain mask for voxels with FA > 0.3. Path tracing will proceed until FA < 0.15 or until the minimum angle between the current and previous path segments >30°. AFQ-derived segmentations of major white matter tracts including the cingulum have been validated in children and adolescents (Yeatman et al., 2012; Ho et al., 2017a, 2020). Importantly, we have used these pre-processing and tractography methods to successfully segment the cingulum tracts in adolescents (Ho et al., 2020), and compute FA as well as other diffusivity metrics (mean diffusivity, axial diffusivity, and radial diffusivity). See Figure 4 for more details.

#### Quantitative MRI

R1 (1/T1), which can be computed from a quantitative MRI (qMRI) scan, has been shown to be a promising complement to DWI for quantifying myelin *in vivo* using MRI (Koenig et al., 1990; Stüber et al., 2014). Moreover, recent evidence suggests that lower R1 is associated with MDD in adults (Sacchet and Gotlib, 2017). We will use a slice-shuffled inversion-recovery simultaneous multi-slice EPI sequence with in-plane acceleration followed by a second scan with reversed phase encoding in order to correct for signal distortion. After running FSL’s *top-up* to estimate and correct for distortions, we will use non-linear least squares modeling to estimate T1 signal per voxel^7^ and then compute the inverse of the result to obtain a voxelwise map of R1 values.

### Inflammatory Cytokines

To measure inflammatory cytokines, we will use a dried blood spot (DBS) protocol. Five blood spots, comprising approximately 150–250 μL per spot, will be collected using mini contact-activated lancets (BD 366594 Microtainer, BD Biosciences, San Jose, CA, United States) to prick the finger after the participant runs their non-dominant hand under hot water for 2 min. Blood spots will be collected on 3 mm filter paper cards (Whatman #903, GE Healthcare, Piscataway, NJ, United States) and will then be dried overnight at room temperature before being transferred to Ziplock bags with a desiccant for storage in a −20°C freezer. All extraction and analysis will take place at the Human Immune Monitoring Center (HIMC) at Stanford University. Prior to analysis, all samples will be extracted, prepared, and diluted 3 fold in the Luminex assay buffer prior to running the 62-plex Luminex assays (eBioscience; San Diego, CA, United States), and then run through a dedicated flow cytometry-based platform, the Luminex FlexMap 3D. The 62-plex Luminex assays use antibody-conjugated bead sets to detect analytes in a multiplexed sandwich immunoassay format. Each bead in the set is identified by a unique spectral barcode of two dyes which is excited by a red laser. The quantity of bound protein will be read via a biotin-conjugated dector antibody bound to streptavidin-phycoerythrin. The streptavidin-phycoerythrin conjugate is excited by the second laser (green). In addition to the 62-plex beads, each well will also contain Assay Chex beads (Radix BioSolutions), which are process control beads that will allow us to normalize the data based on potential confounds (e.g., non-specific binding; Maecker et al., 2020). All data will be analyzed using MasterPlex software (Hitachi Software Engineering America Ltd., MiraiBio Group). Although both median fluorescence intensity (MFI) and calculated concentration values (in pg/mL) will be reported for each analyte, based on prior work demonstrating advantages of using MFI over concentration values for low abundant analytes (Breen et al., 2016), we will conduct all statistical analyses using MFI values. Although we are able to obtain more than 60 analytes using the Luminex assays, to minimize multiple comparisons and pursue hypothesis-driven analyses based on prior literature relating inflammatory cytokines with depression (Howren et al., 2009; Liu et al., 2012; Raison et al., 2013; Valkanova et al., 2013), we will focus on interleukin (IL)-1β, IL-6, IL-10, and tumor necrosis factor (TNF)-α, as these cytokines have also been shown to be assayed reliably from DBS (Miller and McDade, 2012; Skogstrand et al., 2012). Critically, the cytokines we will assay using the described DBS protocol have been validated against plasma obtained through a venipuncture from the same individuals collected at the same time using standard protocols from the field (*R*^2^ = 0.82; Rosenberg-Hasson et al., 2014).

### Statistical Approach and Power Analyses

In this proposed longitudinal study, 60 depressed adolescents will be repeatedly assessed over a considerably long period, allowing for reasonable inference regarding the longitudinal relationships among inflammation, Glu, and ACC connectivity. The core analysis strategy in this project is longitudinal mixed effects modeling (Raudenbush and Bryk, 2002; Singer et al., 2003), where we fully utilize the repeatedly measured primary outcomes of neurophenotypes (i.e., ACC connectivity). For all mixed effects models, maximum likelihood (ML) estimation will be used, which will allow individuals who have outcome data at one or more assessment points to be included in our final analysis.

First, we will employ standard correlation and linear regression analyses to test linear associations among baseline measurements of inflammation, ACC Glu, ACC GSH, ACC Asc, and depression severity, which will provide important insights regarding the relationships across these key variables. Based on the medium effect sizes (*r* = 0.45–0.75) reported in the literature on associations between pro-inflammatory cytokine levels and depression (Liu et al., 2012), between GSH and depression in a pilot study of 11 adults with and 10 adults without depression (Lapidus et al., 2014), and in our pilot data in an independent sample of 22 adolescents with familial risk of mood disorders with elevated depression symptoms who do not meet criteria for MDD (*r* = 0.31), the estimated power to detect a significant association between all our key variables will range from **0.65 to 0.90** with *N* = 60.

To test the hypothesis of our primary aim that glutamate concentrations in the ACC mediate the associations between higher levels of pro-inflammatory cytokines and depressive neurophenotypes (e.g., cingulum FA), we will conduct separate (one for each outcome) mediation models to test whether the effects of T1 cytokines on longitudinal trajectories in these depressive phenotypes are mediated through early changes (changes between T1 and T2) in ACC Glu. We will follow the eligibility and analytical criteria of MacArthur approach for mediator analysis (Kraemer et al., 2002;,  Kraemer and Gibbons 2009), which will be examined by two tests: (1) whether T1 cytokines are significantly correlated with early changes (change between T1 and T2) in ACC Glu (eligibility criteria for mediators); and (2) whether early changes in ACC Glu are significantly correlated with longitudinal trajectories (i.e., estimated slopes) of depressive neurophenotypes (analytical criteria for mediators). Previous studies have documented moderately strong associations between Glu and inflammatory cytokines (Sanacora et al., 2012) as well as between Glu and MDD (Moriguchi et al., 2019); however, none have related Glu in ACC with cytokines specifically and no studies have examined whether changes in Glu are associated with longitudinal trajectories in neurophenotypes (i.e., with resting-state or white matter connectivity). Thus, using a conservative effect size of *r* = 0.4 for both tests, assuming 15% missing data by T3, and conservatively assuming ICC = 0.7 for ACC connectivity based on previous longitudinal MRI studies (Bonekamp et al., 2007; Thomason et al., 2011), we estimate that the power to analytically identify ACC Glu as a mediator is **0.79**. Because of the absence of data in the literature on whether GSH and/or Asc are potential moderators of associations among inflammation, Glu and depressive neurophenotypes, we will focus on hypothesis generation and clinical significance (effect sizes) for the second aim of our study.

Finally, we will use linear models to compare recurrence and remission status by T3 from T1 levels of cytokines, GSH and Asc in ACC, and Glu in ACC. We will also use the intercepts and slopes of ACC connectivity that are estimated from our mixed effects models to compare the groups on longitudinal trajectories of neurophenotypes. Because there are no studies to date comparing trajectories of ACC connectivity in either adolescents or adults with recurrent MDD with recovered counterparts, we estimated power according to effect sizes reported in changes in depressive symptoms between these two groups in adults (*r* = 0.68–0.78; Vittengl et al., 2007). Under these assumptions, and assuming 15% attrition by T3 (*N* = 51), the estimated power to detect group differences is **0.87**.

## Discussion

In summary, in the TIGER study we are comprehensively assessing neurobiological phenotypes of MDD and identifying predictors of recurrence of depression in adolescents. The main aims of this longitudinal study are three-fold: (1) to determine whether glutamate in the ACC mediates the associations between elevated pro-inflammatory cytokines and depressive neurophenotypes; (2) to examine whether antioxidants, such as glutathione and ascorbate, moderate the associations among ACC glutamate concentrations, peripheral levels of inflammation, and longitudinal trajectories of ACC connectivity; (3) to identify neurobiological and clinical predictors of the recurrence of depression over 18 months.

This study is novel in its multimodal approach of examining stress-related mechanisms that contribute to the development and persistence of depression in adolescents. By identifying predictors and mechanisms of the recurrence of depression in adolescents, we are taking an important initial step in generating subtypes or biotypes (i.e., scientifically informed neural subtypes) of depression that will elucidate our understanding of the phenomenology of depression and inform treatment strategies. For example, it is likely that not all depressed adolescents exhibit heightened inflammation or ACC glutamate (relative to healthy controls); those who do may represent a distinct subtype or biotype of depression that does not respond well to first-line treatments. Although TIGER is not a treatment study, we will be able to explore such possibilities, which may inform future research seeking to examine or monitor the efficacy of novel treatments for adolescent depression.

Moreover, given the heterogeneity in subject selection, sample size, scan sequence parameters, field of strength of scanner, and voxel location in previous studies using MRS to examine neurometabolites implicated in depression (Yüksel and Öngür, 2010; Luykx et al., 2012; Moriguchi et al., 2019), a major strength of the multimodal neuroimaging approach of TIGER is that it allows us to integrate information from multiple neuroimaging modalities (e.g., fMRI, diffusion MRI) to enable us to reconcile these potential discrepancies in the literature and gain a more precise understanding of glutamatergic abnormalities underlying MDD (Salvadore and Zarate, 2010; Mathews et al., 2012). Finally, because of the comprehensive clinical and neurobiological data we will be collecting, we will also be in a position to explore additional hypotheses and projects spanning topics outside the main aims of the study, including identifying correlates and predictors of STBs, NSSI behaviors, and examining the contribution of the type and timing of stress to the onset, course, and neurophenotypes of depression.

Although our study has a number of strengths, it also has important limitations. First, we expect some attrition by the end of this longitudinal study, which may reduce statistical power to test all study aims. While we explicitly assumed 15% attrition by the end of the study in our power analyses and are utilizing mixed effects modeling which can account for some missing data (Little and Rubin, 2002), unanticipated events (i.e., COVID-19 pandemic) may further reduce our statistical power. Thus, larger investigations will be necessary to replicate our findings before they can be translated to clinical practice. Second, the age range for inclusion in our study is fairly broad. We selected this age range based on epidemiological rates of the onset of depression (Breslau et al., 2017) while seeking to maximize feasibility in recruitment. Moreover, because we will be assessing adolescents across a relatively wider age range, we will be positioned to model age-related associations in ACC connectivity across the study sample that will allow us estimate patterns of typical and atypical (i.e., depression) trajectories. Nevertheless, it will be important for future investigations to consider recruiting a sample with minimal variability in age in order to better understand the effects of stress-related inflammation and glutamate on neurodevelopment. Third, data collection will occur primarily in laboratory-based settings, which may reduce the generalizability of our study results with respect to the severity and range of symptom profiles, given that a minimum level of functioning is necessary for attending in-lab assessments. Future investigations that rely on naturalistic and easily acquired data are needed to generate scalable markers of behavior and cognition that are neurobiologically validated in order to facilitate the widespread identification of depression (including subtypes or biotypes) in adolescents. Fourth, there are also potential circadian effects on inflammatory markers and neurometabolite concentrations that also need to be considered, particularly in light of compelling evidence of disturbances of sleep and circadian biology in depression (Clarke and Harvey, 2012; Orchard et al., 2020; Walker et al., 2020). While we will be collecting time of day for each measure we collect, which can be used as covariates in sensitivity analyses, future work will be needed to more comprehensively assess circadian rhythms, as well as sleep chronotype and other related factors, on these variables and their processes. Finally, although we are including adolescents who have subthreshold depression according to DSM criteria, dimensional frameworks that conceptualize and characterize the heterogeneity of symptom presentation found in mood and anxiety disorders more broadly (e.g., RDoC and HiTOP; Cuthbert and Insel, 2013; Kotov et al., 2017) are particularly promising approaches to generating novel brain-based models that can lead to the effective treatment – and possibly prevention – of these debilitating conditions.

In conclusion, the TIGER project will collect comprehensive neurobiological, clinical, and cognitive data to assess the associations between inflammatory and glutamatergic pathways to predict the recurrence of depression in adolescents. The ultimate aim of the TIGER project is to set the foundation for brain-based models of depression in adolescents by elucidating neurobiological mechanisms that facilitate risk identification and treatment decisions, and by informing research focused on the development of novel treatment targets for this particularly vulnerable population.

## Ethics Statement

The studies involving human participants were reviewed and approved by Stanford University Institutional Review Board and University of California, San Francisco Institutional Review Board. Participants under the age of 18 provided written informed assent and their parent(s)/legal guardian(s) provided written informed consent on their behalf. Participants 18 and older provided written informed consent.

## Author Contributions

TH, IG, MS, MG, DS, YR-H, and HM designed the study. TH obtained funding for the study. JW, GT, RW, JS, and TH wrote the first draft of the manuscript. All authors contributed to the writing of the manuscript.

## Conflict of Interest

The authors declare that the research was conducted in the absence of any commercial or financial relationships that could be construed as a potential conflict of interest.

## Footnotes

1 https://www.phenxtoolkit.org/sub-collections/view/3

2 http://surfer.nmr.mgh.harvard.edu/fswiki/recon-all

3 http://enigma.ini.usc.edu/protocols/imaging-protocols/

4 https://cni.stanford.edu/wiki/Spectro#Automated_magnetic_resonance_spectroscopy_voxel_placement_tools

5 http://vistalab.stanford.edu/software

6 https://github.com/jyeatman/AFQ

7 https://github.com/cni/t1fit/blob/master/t1_fitter.py
